# Apoptosis Effects of Dihydrokaempferol Isolated from* Bauhinia championii* on Synoviocytes

**DOI:** 10.1155/2018/9806160

**Published:** 2018-12-02

**Authors:** Yuqin Zhang, Guohong Yan, Chengtao Sun, Huang Li, Yanhui Fu, Wei Xu

**Affiliations:** ^1^Pharmacy College of Fujian University of Traditional Chinese Medicine, Shangjie Minhou, Fuzhou, Fujian, China; ^2^Key Laboratory of Tropical Medicinal Plant Chemistry of Ministry of Education, Hainan Normal University, Haikou, China; ^3^State Key Laboratory of Chinese Pharmacies of Fujian Provincial, Department of Science and Technology of Fujian University of Traditional Chinese Medicine, Shangjie Minhou, Fuzhou, Fujian, China; ^4^People's Hospital Affiliated to Fujian University of Traditional Chinese Medicine, Bayiqi Road, Fuzhou, Fujian, China

## Abstract

*Bauhinia championii *(Benth.) Benth. is a traditional medicinal plant used in China to treat rheumatoid arthritis (RA), especially in She ethnic minority group. This study focused on the active constituents from the rattan of* B. championii *(Benth.) Benth., which possess potential apoptosis effects. A conventional phytochemical separation method for the isolation of compounds from the ethyl acetate extract of* B. championii* was developed. The procedure involved extraction, liquid–liquid partitioning with ethyl acetate, and subsequent compound purification, respectively. Additionally, cell viability of dihydrokaempferol found abundantly in it was evaluated* in vitro* by MTS, and the antiapoptosis effect was evaluated by annexin V/PI staining (Flow Cytometry Analysis) and western blot. The results showed that nine flavonoids, and five other compounds, were isolated from the ethyl acetate extract of* B. championii* and were identified as *β*-sitosterol (1), 5,6,7,3',4',5'-hexamethoxyflavone (2), 3',4',5,7-tetrahydroxyflavone (3), 5,7,3',4',5'-pentamethoxyflavone (4), 4'-hydroxy-5,7,3',5'-pentamethoxyflavone (5), apigenin (6), liquiritigenin (7), 5, 7-dihydroxylcoumarin (8), 3',4',5,7, -pentamethoxyflavone (9),* n*-octadecanoate (10), lupine ketone (11), dibutylphthalate (12), dihydrokaempferol (13), and 5,7,3′,5′-tetrahydroxy-6-methylflavanone (14). Among these compounds, 5-14 were isolated for the first time from* B. championii. *In addition, apoptosis effects of abundant dihydrokaempferol were evaluated* in vitro*. Dihydrokaempferol exhibited inhibitory effects on the proliferation of synoviocytes. Furthermore, dihydrokaempferol promoted Bax and Bad expression, as well as the cleavage of caspase-9, caspase-3, and PARP. Meanwhile, it inhibited Bcl-2 and Bcl-xL expression. These findings indicate that dihydrokaempferol isolated from the ethyl acetate extract of* B. championii* effectively promotes apoptosis, which is an important process through suppression of apoptotic activity. The results are encouraging for further studies on the use of* B. championii* in the treatment of RA.

## 1. Background

Rheumatoid arthritis (RA) is a systemic autoimmune disease characterized by systemic inflammation, persistent synovitis, and autoantibodies, leading to joint distortion and loss of function [1–3]. RA is a common chronic arthritis, affecting 1% of the world's population [4], but, unfortunately, current treatment (nonsteroidal anti-inflammatory drugs, biological agents, disease-modifying antirheumatic drugs, and so on) only slows the progression of RA rather than prevent it, and it also causes several severe side effects, such as bone loss, dermatological damage, anaemia, and neutropenia [4]. RA not only seriously affects the patients' quality of life, but also gives the family and society a heavy burden. So, effective, safe test and economic drugs and therapeutic strategy are desired.

Chinese medicine is a treasure of China, in recent decades, occupying an increasingly important position in the pharmaceutical industry. The novel structures are extracted from Chinese herbal medicine, then having fewer side effects, attracting more and more researchers to develop Chinese herbal medicine.

*B. championii* is a perennial vine plant of legume, widely distributed in the southeast coast of China. It is a traditional folk medicine which can be used for expelling wind, promoting blood circulation, relieving blood circulation, and treating rheumatoid arthritis [5, 6]. It was reported that polysaccharides [7], flavonoids [8, 9], and alkaloids [10] extracted from* B. championii* (Benth.) Benth had a strong physiological activity [11, 12]. Our preliminary results [13, 14] showed that 90% EtOH extract of* B. championii* (Benth.) Benth showed a significant antiproliferative effect on synovial cells* in vitro*. However, the material basis behind* B. championii*-mediated mitigation of RA pathogenesis has not yet been investigated.

In this study, we aimed to report compounds isolated from the extract of ethyl acetate. In addition, the antiproliferative effect of isolated compounds was evaluated and further explored the action mechanisms of dihydrokaempferol.

## 2. Methods

### 2.1. General Experimental Procedures

Optical rotations were measured with a JASCO P-1020 digital polarimeter (JASCO Corporation, Tokyo, Japan). UV spectra were recorded on a Beckman DU 640 spectrophotometer (Beckman Coulter, Inc., Miami, USA). IR spectra were recorded on a Nicolet Nexus 470 spectrophotometer (Thermo Fisher Scientific Inc., Massachusetts, USA) in KBr discs. HR-ESI-MS spectra were measured on a Micromass Q-TOF Ultima Global GAA076 LC mass spectrometer (waters corporation, Milford, USA). NMR spectra were recorded on Bruker 400 MHz spectrometers (Bruker Daltonics Inc., Karlsruhe, Germany) using TMS (Tetramethyl silane) as an internal standard* δ*. X-ray diffraction data were collected on an Agilent Technologies Gemini A Ultra system (Agilent Technologies Inc., Palo Alto, USA). Semipreparative HPLC was performed on an Agilent 1260 LC series with a DAD detector using an Agilent Eclipse XDB-C_18_ column (250 × 10.0mm, 5 *µ*m). Silica gel (300-400 mesh, Qingdao Marine Chemical Inc., China), Silica gel (200-300) mesh (Qingdao Marine Chemical Inc., China), Silica gel H (10-40 *μ*m, Qingdao Marine Chemical Inc., China), Lichroprep RP-18 gel (40-63 *μ*m, Merck, Darmstadt, Germany), and Sephadex LH-20 (40-70 *μ*m, Amersham Biosciences, Sweden) were used for column chromatography (CC).

### 2.2. Plant Material

Rattans of* B. championii *(Benth.) Benth were collected in the mountain woods of Minhou (E119.22, N25.88, Fuzhou, China), in April 2011, and identified by Prof. Lu Wei (Fujian University of Traditional Chinese Medicine, China). A voucher specimen (No. BC201104) has been deposited in College of Pharmacy of Fujian University of Traditional Chinese Medicine, Fujian 350122, China.

### 2.3. Extraction and Isolation

We followed the methods of Wu et al. (2018) to perform the extraction and isolation [15]. The powdered air-dried rattans of* B. championii *(Benth.) Benth (63.0 kg) were extracted with 90% EtOH at room temperature for three times, each for 3 days. The solvent was combined and condensed* in vacuum* to yield a crude extract (2650 g). After being suspended in water (8.0 L), the crude extract was applied to a liquid–liquid partitioning against petroleum ether (8.0 L3), EtOAc (8.0 L3), continuously and obtained petroleum ether extract and EtOAc extract. The EtOAc extract (435.0 g) was dried and chromatographed over silica gel eluting with a gradient of CHCl_3_/CH_3_OH (100:0 to 0: 100, v/v) to yield five fractions (Fr.1–Fr.6). Fraction 1 (21.4 g) was subjected to column chromatography (CC) on silica gel using PE/acetone (50:1 to 1:50, v/v) to afford five subfractions (1A–1E). Subfraction 1C (4.2 g) was subjected to purified by Sephadex LH-20 eluted with CH_3_OH to afford compound** 12 **(9.6 mg). Subfraction 1D (3.6 g) was subjected to chromatograph over Sephadex LH-20 gel column eluted with CHCl_3_/CH_3_OH (2:3, v/v) to give compound** 11** (11.7 mg). Fraction 2 (46.8 g) was subjected to RP-18 using CH_3_OH/H_2_O (20:80 to 100:0, v/v) to afford six subfractions 2A–2F. Fraction 2B (4.6 g) was subjected to CC on silica gel using PE/acetone (50:1 to 1:50, v/v) and further purified by Sephadex LH-20 gel column eluted with CH_3_OH to afford compounds** 3 **(12.5 mg),** 4 **(6.3 mg), and** 5 **(5.2 mg). Fraction 2C (6.8 g) was subjected to CC on silica gel eluted with PE/acetone (50:1 to 1:50, v/v) then separated by Sephadex LH-20 column chromatography eluted with CH_3_OH to yield compounds** 2 **(3.8 mg) and** 6** (4.1 mg). Fraction 2C was separated by semipreparative HPLC (Waters XBridge C18 column, i.d. 250 × 10 mm, 5 *μ*m, 68% CH_3_CN with 0.5% v/v CH_3_COOH, 3.0 mL/min, t_R_ 16.5, and 22.0 min) to afford compounds** 1 **(4.6 mg) and** 10** (5.4 mg). Compounds** 7 **(7.4 mg),** 8** (6.2 mg), and** 9** (8.5 mg) were obtained, respectively, from fraction 2D (5.4 g) by Sephadex LH-20 column chromatography eluted with CH_3_OH and then separated by semipreparative HPLC (Waters XBridge C18 column, 250 × 10 mm, 5 *μ*m, 62% CH_3_CN, 3.0 mL/min, t_R_ 10.8, 13.6 and 18.7 min). Fraction 3 (108.9 g) was subjected to CC on silica gel eluted with PE/acetone (50:1 to 1:50, v/v) to afford six subfractions 3A–3F. Fraction 3A (488 mg) was separated by semipreparative HPLC (Waters XBridge C_18_ column, i.d. 250 × 10 mm, 5* μ*m, 45% MeOH, 3.0 mL/min, *t*_R_ 13.8 min) to give compound** 12 **(66.8 mg). Compound** 14** (23.8 mg) was obtained from fraction 3B (540 mg) by semipreparative HPLC (Waters XBridge C_18_ column, i.d. 250 × 10 mm, 5* μ*m, 50% MeOH, 3.0 mL/min, t_R_ 22.3 min). According to previous cell activity screening results (unpublished data), dihydrokaempferol (compound 13) was selected for our subsequent experiments.

### 2.4. Cells Culture

Rheumatoid arthritis fibroblast-like synoviocytes (RA-FLSs) were isolated and cultured from synovium tissue of Wistar rats with rheumatoid arthritis induced by collagen the same as previous studies [14]. In brief, synovium tissues were isolated and digested enzymatically by 0.2% collagen type II (Sigma) in RPMI 1640 for 2 h at 37°C. The obtained cell suspension was plated onto culture dishes and cells were used for subsequent experiments between passages 3 and 6.

### 2.5. Cell Viability Assay

RA-FLSs were cultured in 96-well plates and the cell viability was assessed by MTS assay. RA-FLSs cultured in 96-well plates were treated with dihydrokaempferol at various concentrations (0.3, 3, 30, 300 *μ*M) for 48 h, followed by incubation with MTS for an additional 4 h at 37°C. Then the absorbance at 570 nm was taken by a microplate reader (Infinite M200 Pro, TECAN).

### 2.6. Flow Cytometric Analysis

RA-FLSs cultured in 6-well plates were treated with dihydrokaempferol at various concentrations for 48 h. Then, cells were harvested and quantitated according to the manufacture's protocol. Briefly, cells were resuspended in binding buffer and were incubated in 5 *µ*L of annexin V-FITC and 5 *µ*L of PI at room temperature for 15 min in the dark. Finally, 400 *µ*L of binding buffer was added and then samples were analyzed by flow cytometer with an excitation wavelength of 488 nm and an emission wavelength of 530 nm (Becton-Dickinson, Bedford, MA, USA). Apoptotic cells were expressed as a percentage of the total number of cells and three times of flow cytometric analysis have been done.

### 2.7. Western Blot Analysis

According to the results of cell viability, western blot analysis was used to evaluate the proteins level affected by dihydrokaempferol. Its method was similar to those described previously [14]. After being treated with dihydrokaempferol, cells were collected and lysed by lysis buffer, then they were centrifuged at 12,000 g for 15 min by Heraeus Sepatech (#5418, Eppendorf China, Shanghai, China). The supernatant was collected and the protein concentration was determined by the BCA method. Then protein mixed with loading buffer and incubated in 100°C for 6 min. Ultimately, samples were analyzed for western blot analysis with primary antibodies to cleaved caspase-3 (1:500), cleaved caspase-9 (1:500), p-Bad (1:500), Bcl-xL (1:1,000), Bax (1:1,000), Bcl-2 (1:1,000), cleaved PARP (1:1,000), and *β*-actin (1:1,000) overnight at 4°C. Finally they were evaluated using an enhanced chemiluminescence detection system (Amersham Biosciences, Piscataway, NJ, USA) and the expression level was analyzed using a Chemidoc XRS imager system (Bio-Rad Laboratories, Hercules, CA, USA).

### 2.8. Statistical Analysis

One-way analysis of variance (ANOVA) (SPSS 20.0 statistical software, Chicago, IL, USA) was used to evaluate multiple group difference.* P *< 0.05 was considered to indicate statistical significance.

## 3. Results

### 3.1. Isolation and Identification of Compounds

**Figure 1:**
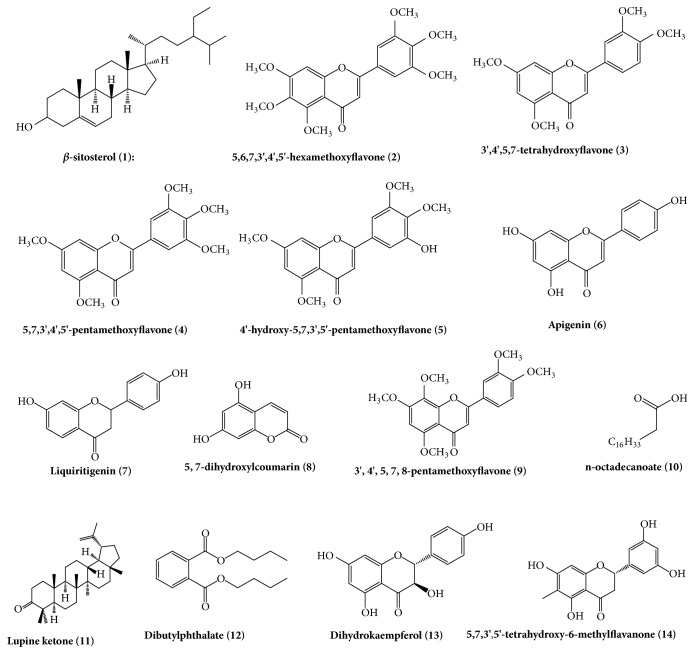
The chemical structures of the compounds isolated from ethyl acetate fraction of* B. championii*.

The 90% EtOH extract of air-dried rattans of* B. championii *(Benth.) Benth was insoluble in water and extracted with petroleum ether and EtOAc. Various fractions were subjected to various chromatographic methods including silica gel, reverse silica gel, LH-20, and semipreparative HPLC to purify and be separated. A total of 13 compounds were obtained, including nine flavonoids and five other compounds. The chemical structures of them were presented in Figure 1.

**β** **-Sitosterol (1)**: white amorphous powder; C_29_H_50_O, ESI-MS* m/z*: 415 [M+H]^+^, 437 [M+Na]^+^. ^1^H-NMR (CDCl_3_, 400 MHz)* δ*: 5.35 (1H, d,* J* = 5.0 Hz, H-6), 3.52 (1H, m, H-3), 1.02 (3H, s, H-19), 0.91 (3H, d,* J* = 7.2 Hz, H-21), 0.85 (3H, t,* J* = 7.2 Hz, H-29), 0.83 (3H, d,* J *= 7.2 Hz, H-26), 0.81 (3H, d,* J* = 7.2 Hz, H-27), 0.68 (3H, s, H-18); ^13^C-NMR (CDCl_3_, 100 MHz)* δ*: 140.7 (C-5), 121.7 (C-6), 71.8 (C-3), 56.7 (C-14), 56.0 (C-17), 50.1 (C-9), 45.8 (C-4), 42.3 (C-13), 42.3 (C-24), 39.7 (C-12), 37.2 (C-1), 36.5 (C-10), 36.1 (C-20), 33.9 (C-7), 31.9 (C-22), 31.9 (C-2), 31.6 (C-8), 29.1 (C-25), 28.2 (C-16), 26.0 (C-28), 24.3 (C-15), 23.0 (C-23), 21.1 (C-11), 19.8 (C-26), 19.4 (C-19), 19.0 (C-27), 18.8 (C-21), 12.8 (C-29), 11.8 (C-18). These data were identical with those of *β*-sitosterol [16].

**5,6,7,3**′**,4**′**,5**′**-Hexamethoxyflavone (2)**: faint yellow amorphous powder, HCl-Mg reaction (+); C_21_H_22_O_8_, ESI-MS* m/z*: 403 [M+H]^+^. ^1^H-NMR (CD_3_OD, 400 MHz)* δ*: 7.24 (2H, s, H-2', H-6'), 7.13 (1H, s, H-3), 6.67 (1H, s, H-8), 4.01 (3H, s, -OCH_3_), 3.94 (6H, s, -OCH_3_), 3.92 (3H, s, -OCH_3_), 3.86 (3H, s, -OCH_3_), 3.84 (3H, s, -OCH_3_); ^13^C-NMR (CD_3_OD, 100 MHz)* δ*: 179.6 (C-4), 163.4 (C-2), 160.1 (C-7), 156.2(C-5), 155.1 (C-5'), 155.1 (C-3'), 153.3 (C-9), 142.5 (C-4'), 141.9 (C-6), 127.9 (C-1'), 113.2 (C-3), 108.2 (C-10), 105.0 (C-2'), 105.0 (C-6'), 98.1 (C-8), 62.6 (-OCH_3_), 61.8 (-OCH_3_), 61.3 (-OCH_3_), 57.1 (-OCH_3_), 57.0 (-OCH_3_), 57.0 (-OCH_3_). These data were identical with those of 5,6,7,3',4',5'-hexamethoxyflavone [17].

**3**′**,4**′**,5,7-Tetrahydroxyflavone (3)**: faint yellow amorphous powder, HCl-Mg reaction (+); C_19_H_18_O_6_, ESI-MS* m/z*: 343 [M+H]^+^, 365 [M+Na]^+^. ^1^H-NMR (CDCl_3_, 400 MHz)* δ*: 7.51 (1H, dd,* J *= 8.4, 2.0 Hz, H-6′), 7.32 (1H, d,* J *= 2.0 Hz, H-2′), 6.96 (1H, d,* J *= 8.4 Hz, H-5′), 6.60 (1H, s, H-3), 6.56 (1H, d,* J *= 2.0 Hz, H-8), 6.38 (1H, d,* J *= 2.0 Hz, H-6), 3.97 (3H, s, 3′-OCH_3_), 3.96 (3H, s, 5-OCH_3_), 3.96 (3H, s, 4′-OCH_3_), 3.92 (3H, s, 7-OCH_3_); ^13^C-NMR (CDCl_3_, 100 MHz)* δ*: 177.6 (C-4), 164.0 (C-7), 160.9 (C-2), 160.6 (C-5), 159.9 (C-9), 151.7 (C-4′), 149.3 (C-3′), 124.1(C-1′), 1l9.5 (C-6′), 111.1 (C-5′), 109.2 (C-10), 108.6 (C-2′), 108.0 (C-3), 96.1 (C-6), 92.9 (C-8), 56.5 (4′-OCH_3_), 56.1 (5-OCH_3_), 56.1 (3′-OCH_3_), 55.8 (7-OCH_3_). These data were identical with those of 5,7,3',4'- tetrahydroxyflavone [18].

**5,7,3**′**,4**′**,5**′**-Pentamethoxyflavone (4)**: faint yellow amorphous powder, HCl-Mg reaction (+); C_20_H_20_O_7_, ESI-MS* m/z*: 373 [M+H]^+^, 395 [M+Na]^+^. ^1^H-NMR (CDCl_3_, 400 MHz)* δ*: 7.10 (1H, s, H-2′, 6′), 6.66 (1H, s, H-3), 6.60 (1H, d,* J *= 2.0 Hz, H-8), 6.42 (1H, d,* J *= 2.0 Hz, H-6), 4.00 (3H, s, 5-OCH_3_), 3.98 (3H, s, 7-OCH_3_), 3.98 (3H, s, 3′-OCH_3_), 3.96 (3H, s, 4′-OCH_3_), 3.95 (3H, s, 5′-OCH_3_); ^13^C-NMR (CDCl_3_, 100 MHz)* δ*: 177.6 (C-4), 164.1 (C-7), 161.0 (C-2), 160.5 (C-5), 159.9 (C-9), 153.6 (C-4′), 140.9 (C-3′), 126.8 (C-1′), 109.2 (C-10), 108.9 (C-5′), 103.4 (C-6′), 103.4 (C-2′), 96.4 (C-6), 93.2 (C-8), 61.1 (4′-OCH_3_),56.5 (5-OCH_3_),56.4 (5′-OCH_3_), 56.4 (3′-OCH_3_), 55.8 (7-OCH_3_). These data were identical with those of 5,7,3',4',5'-pentamethoxyflavone [19].

**4**′**-Hydroxy-5,7,3**′**,5**′**-pentamethoxyflavone (5)**: faint yellow amorphous powder, HCl-Mg reaction (+); C_19_H_18_O_7_, ESI-MS* m/z*: 359 [M+H]^+^, 381 [M+Na]^+^. ^1^H-NMR (CDCl_3_, 400 MHz)* δ*: 7.12 (1H, s, H-2′, 6′), 6.67 (1H, s, H-3), 6.58 (1H, d,* J *= 2.0 Hz, H-8), 6.40 (1H, d,* J *= 2.0 Hz, H-6), 3.98 (3H, s, 7-OCH_3_), 3.98 (3H, s, 3′-OCH_3_), 3.96 (3H, s, 5-OCH_3_), 3.95 (3H, s, 5′-OCH_3_). These data were identical with those of 4'-hydroxy-5,7,3',5'-pentamethoxyflavone [20].

**Apigenin (6)**: faint yellow amorphous powder, HCl-Mg reaction (+); C_15_H_10_O_5_, ESI-MS* m/z*: 271 [M+H]^+^. ^1^H-NMR (DMSO-*d*_*6*_, 400 MHz)* δ*: 12.99 (1H, s, 5-OH), 12.42 (1H, s, 4'-OH), 10.36 (1H, s, 7-OH), 7.92 (2H, d,* J *= 8.8 Hz, H-2′, 6′), 6.94 (2H, d,* J *= 8.8 Hz, H-3′, 5′), 6.77 (1H, s, H-3), 6.49 (1H, d,* J *= 2.0 Hz, H-8), 6.21 (1H, d,* J *= 2.0 Hz, H-6); ^13^C-NMR (DMSO-*d*_*6*_, 100 MHz)* δ*: 181.7 (C-4), 164.2 (C-2), 163.7 (C-7), 161.4 (C-9), 161.1 (C-4'), 157.3 (C-5), 128.4 (C-2', 6'), 121.2 (C-1'), 115.9 (C-3', 5'), 103.6 (C-10), 102.8 (C-3), 98.8 (C-6), 93.9 (C-8). These data were identical with those of apigenin [21].

**Liquiritigenin (7)**: faint yellow amorphous powder, HCl-Mg reaction (+); C_15_H_12_O_4_, ESI-MS* m/z*: 257 [M+H]^+^. ^1^H-NMR (CD_3_OD, 400 MHz)* δ*: 7.73 (1H, d,* J *= 8.5 Hz, H-5), 7.32 (2H, d,* J *= 8. 5 Hz, H-2', 6'), 6.84 (2H, d,* J *= 8.5 Hz, H-3', 5'), 6.51 (1H, dd,* J *= 8.5, 2.5 Hz, H-6), 6.36 (1H, d,* J *= 2.0 Hz, H-8), 5.34 (1H, dd,* J* = 13.5, 3.0 Hz, H-2), 3.03 (1H, dd,* J *= 17.0, 13.5 Hz, H-3*α*), 2.68 (1H, dd,* J *= 17.0, 3.0 Hz, H-3*β*); ^13^C-NMR (CD_3_OD, 100 MHz)* δ*: 193.6 (C-4), 167.3 (C-7), 165.6 (C-9), 158.9 (C-4'), 131.4 (C-1'), 129.9 (C-5), 129.0 (C-2', 6'), 116.4 (C-3', 5'), 114.8 (C-10), 112.0 (C-6), 104.0 (C-8), 81.0 (C-2), 45.0 (C-3). These data were identical with those of liquiritigenin [22].

**5, 7-Dihydroxylcoumarin (8)**: faint yellow needle crystal (MeOH); C_9_H_6_O_4_; ESI-MS* m/z *178 [M+H]^+^. ^1^H NMR (DMSO-*d*_*6*_, 400 MHz)* δ*: 7.95 (1H, d,* J = *9.4 Hz, H-4), 6.01 (1H, d,* J = *9.4 Hz, H-3), 6.24 (1H, s, H-6), 6.16 (1H, s, H-8); ^13^C NMR (DMSO-*d*_*6*_, 100 MHz)* δ*: 162.2 (C-7), 160.8 (C-5), 156.5 (C-9), 156.2 (C-2), 139.6 (C-4), 108.4 (C-3), 101.7 (C-10), 98.2 (C-6), 93.9 (C-8). All the above data were in good agreement with those of 5,7-dihydroxyl coumarin [23].

**3**'**,4**'**,5,7,8-Pentamethoxyflavone (9)**: faint yellow amorphous powder, HCl-Mg reaction (+); C_15_H_12_O_4_, ESI-MS* m*/*z*: 372 [M+H]^+^. ^1^H-NMR (DMSO-*d*_*6*_, 400 MHz)* δ*: 7.57 (1H, dd,* J *= 8.5, 2.0 Hz, H-6′), 7.45 (1H, d,* J *= 2.0 Hz, H-2′), 7.06 (1H, d,* J *= 8.7 Hz, H-5′), 7.06 (1H, s, H-6), 6.60 (1H, s, H-3), 4.00 (3H, s, 5-OCH_3_), 3.93 (3H, s, 7-OCH_3_), 3.92 (3H, s, 3′-OCH_3_), 3.90 (3H, s, 4′-OCH_3_), 3.86 (3H, s, 5′-OCH_3_); ^13^C-NMR (DMSO-*d*_*6*_, 100 MHz)* δ*: 179.6 (C-4), 163.8 (C-7), 160.0 (C-2), 156.1 (C-5), 153.9 (C-9), 153.3 (C-4′), 150.9 (C-3′), 141.8 (C-1′), 124.9 (C-6′), 121.2 (C-5′), 113.1 (C-10), 112.8 (C-3), 110.5 (C-2′), 107.1 (C-6), 98.0 (C-8), 61.1 (4′-OCH_3_), 56.4 (5-OCH_3_), 56.3 (5′-OCH_3_), 56.3 (3′-OCH_3_), 55.7 (7-OCH_3_). All the above data were in good agreement with those of 3',4',5,7,8-pentamethoxyflavone [24].

***n*-Octadecanoate (10)**: white waxy powder, C_18_H_36_O_2_, ESI-MS* m*/*z*: 285 [M+H]^+^. ^1^H-NMR (CDCl_3_, 400 MHz)* δ*: 2.35 (2H, t,* J *= 7.6 Hz, H-2), 1.63 (2H, m, H-2), 1. 25 (28H, m), 0.88 (3H, t,* J *= 7. 2 Hz, H-24); ^13^C-NMR (CDCl_3_, 100 MHz)* δ*: 179. 6 (C-1), 34.0 (C-2), 24.7 (C-3), 29.7 (7C), 29.6 (C-19), 29.5 (C-20), 29.4 (C-19), 29.3 (C-20), 29.1 (C-21), 32.0 (C-22), 22.7 (C-23), 14.1 (C-24). All the above data were in good agreement with those of* n*-octadecanoate [25].

**Lupine ketone (11)**: white amorphous powder; C_30_H_48_O, ESI-MS* m/z*: 425 [M+H]^+^, 447 [M+Na]^+^. ^1^H-NMR (CDCl_3_, 400 MHz)* δ*: 4.69 (1H, br s, H-29*a*), 4.57 (1H, br s, H-29*b*), 1.68, 1.07, 1.06, 1.03, 0.96, 0.93, 0.80 (3H×7, s, CH_3_×7); ^13^CNMR (CDCl_3_, 100 MHz) *δ*_*C*_: 218.0 (C-3), 150.8 (C-20), 109.4 (C-29), 54.9 (C-5), 49.8 (C-9), 48.3 (C-18), 47.9 (C-19), 47.3 (C-4), 43.0 (C-17), 42.9 (C-14), 40.8 (C-8), 40.0 (C-22), 39.6 (C-1), 38.2 (C-13), 36.9 (C-10), 35.5 (C-16), 34.1 (C-2), 33.6 (C-7), 29.8 (C-21), 27.4 (C-15), 26.7 (C-23), 25.2 (C-12), 21.5 (C-11), 21.0 (C-24), 19.7 (C-6), 19.3 (C-30), 18.0 (C-28), 16.0 (C-25), 15.8 (C-26), 14.5 (C-27). These data were identical with those of lupine ketone [26].

**Dibutylphthalate (12)**: white oil, EI-MS* m/z *279 [M+H]^+^. ^1^H NMR (CDCl_3_, 400 MHz)* δ*: 7.75 (2H, br,* J *= 6.7 Hz, H-3, 6), 7.55 (2H, m, H-4, 5), 4.33 (2H×2, t,* J *= 7.0 Hz, H-1', C-1”), 1.74 (2H×2, tt,* J *= 7.0 Hz, H-2', C-2”), 1.45 (2H×2, m, H-3', C-3”), 0.96 (3H×2, t,* J *= 7.0 Hz, CH_3_-4', C-4”); ^13^C NMR (CDCl_3_, 100 MHz)* δ*: 13.5 (C-4', C-4”), 19.2 (C-3', C-3”), 30.6 (C-2', C-2”), 65.8 (C-1', C-1”), 128.5 (C-3 and C-6), 130.7 (C-4 and C-5), 132.2 (C-1 and C-2), 167.4 (C-7 and C-7'). These data were identical with those of dibutylphthalate [27].

**Dihydrokaempferol (13)**: faint yellow amorphous powder, HCl-Mg reaction (+); C_15_H_12_O_6_, ESI-MS* m/z*: 311 [M+H]^+^. ^1^H-NMR (DMSO-*d*_*6*_, 400 MHz)* δ*: 11.95 (1H, s, OH-5), 7.33 (2H, d,* J *= 8.4 Hz, H-2′, 6′), 6.81 (2H, d,* J *= 8.4 Hz, H-3′, 5′), 5.92 (1H, d,* J *= 2.0 Hz, H-8), 5.87 (1H, d,* J *= 2.0 Hz, H-6), 5.06 (1H, d,* J *= 11.4 Hz, H-2), 4.59 (1H, d,* J *= 11.4 Hz, H-3); ^13^C-NMR (DMSO-*d*_*6*_, 100 MHz)* δ*: 197.7 (C-4), 167.1 (C-7), 163.3 (C-5), 162.6 (C-9), 157.7 (C-4′), 129.4 (C-1′), 127.6 (C-2′, 6′), 114.9 (C-3′, 5′), 100.3 (C-10), 96.1 (C-6), 95.1 (C-8), 82.9 (C-2), 71.4 (C-3). These data were identical with those of dihydrokaempferol [28].

**5,7,3**′**,5**′**-Tetrahydroxy-6-methylflavanone (14)**: faint yellow amorphous powder, HCl-Mg reaction (+); C_16_H_12_O_6_, ESI-MS* m/z*: 301[M+H]^+^. ^1^H-NMR (DMSO-*d*_*6*_, 400 MHz)* δ*: 6.82 (1H, s, H-5′), 6.73 (1H, d,* J *= 8.0 Hz, H-2′), 6.66 (1H, d,* J *= 8.0 Hz, H- 6′), 5.99 (1H, s, H-8), 5.10 (1H, dd,* J *= 12.0, 3.0 Hz, H-2), 2.94 (1H, dd,* J *= 16.8, 12.0 Hz, H-3*α*), 2.56 (1H, dd,* J *= 16.8, 3.0 Hz, H-3*β*), 1.75 (3H, s, 6-CH_3_); ^13^C-NMR (DMSO-*d*_*6*_, 400 MHz)* δ*: 196.0 (C-4), 164.7 (C-7), 160.4 (C-5), 160.0 (C-9), 145.1 (C-3′), 144.6 (C-5′), 129.7 (C-1′), 118.2 (C-6′), 115.4 (C-2′), 114.0 (C-4′), 103.7 (C-6), 101.2 (C-10), 94.3 (C-8), 78.1 (C-2), 41.6 (C-3), 6.6 (6-CH_3_). These data were identical with those of 5,7,3′,5′- tetrahydroxy-6-methylflavanone [29].

### 3.2. Dihydrokaempferol Decreases the Proliferation of RA-FLSs

**Figure 2:**
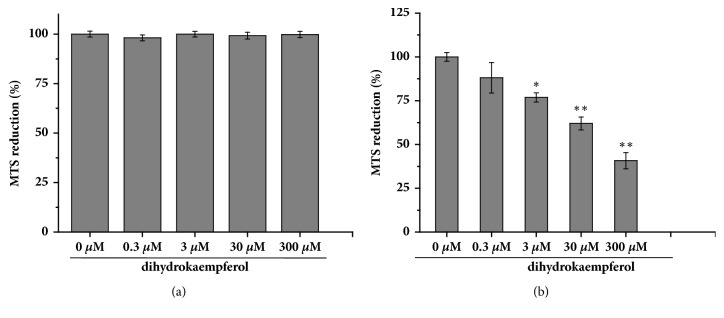
Dihydrokaempferol inhibits the viability of RA-FLSs. (a) The effect of dihydrokaempferol itself on basal growth in FLSs. FLSs were treated with different concentrations of dihydrokaempferol (0.3, 3, 30, 300 *μ*M) for 48 h, then cell viability was assessed by MTS assay. Data are represented as mean ± SD, n = 6 wells for each group. (b) RA-FLSs were treated with different concentrations of dihydrokaempferol (0.3, 3, 30, 300 *μ*M) for 48 h, then cell viability was assessed by MTS assay. Data are represented as mean ± SD, n = 6 wells for each group. ^*∗*^p < 0.05 and ^*∗∗*^p < 0.01 versus the control group.

As illustrated in Figure 2, dihydrokaempferol (0.3, 3, 30, 300 *μ*M) had no significant effect of cell survival on normal synoviocytes (Figure 2(a)). But dihydrokaempferol (0.3, 3, 30, 300 *μ*M) concentration dependently decreased the viability of RA-FLSs (Figure 2(b)). Treatment of these cells with more than 3 *µ*M concentration of dihydrokaempferol for 48 h resulted in significant decrease of cell viability (Figure 2(b)). In light of these findings, we used 3, 30 *µ*M concentration of dihydrokaempferol for our subsequent experiments.

### 3.3. Dihydrokaempferol Induces Apoptosis in RA-FLSs

**Figure 3:**
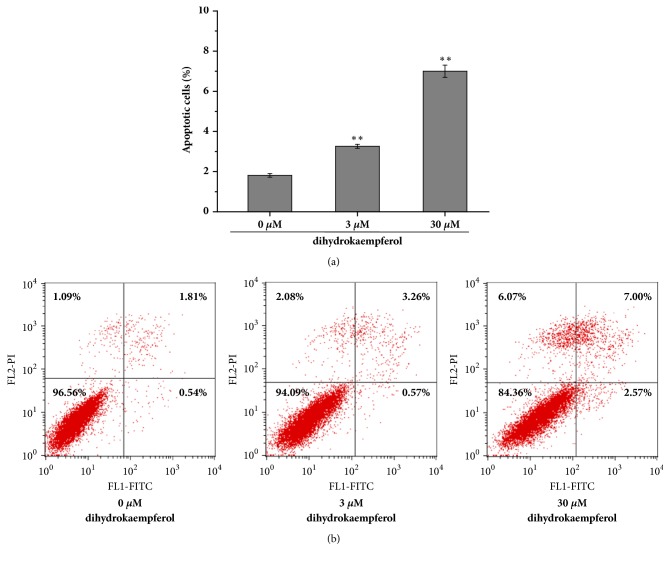
Dihydrokaempferol induces apoptotic death in RA-FLSs. Measurement of apoptosis was used by annexin V/PI staining (Flow Cytometry Analysis). Representative dot plots of flow cytometry are shown in upper panels.

To confirm whether dihydrokaempferol induced apoptosis in RA-FLSs, the annexin V/PI double staining assay was performed. It examined the reversion of phosphatidylserine (a marker for apoptosis) by flow cytometric analysis. As demonstrated in Figure 3, following treatment with dihydrokaempferol (3, 30 *µ*M), the percentage of apoptotic cells (including early and late apoptotic cells) was found gradually increased (~3.8% and ~9.6%, respectively) as compared to control treatment (~2.3%). It was suggested that dihydrokaempferol significantly induced apoptosis in RA-FLSs.

### 3.4. Dihydrokaempferol Regulated the Protein Expression of Apoptosis in RA-FLSs

**Figure 4:**
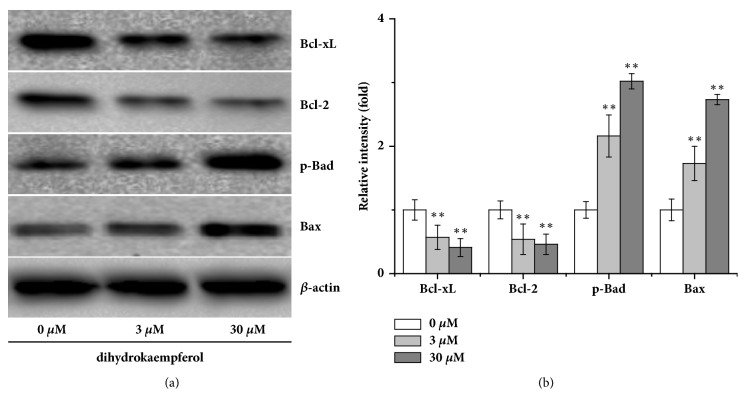
Dihydrokaempferol promoted Bax and Bad expression and inhibited Bcl-2 and Bcl-xL expression. (a) Representative western blot and (b) the relative optical densities analysis of the levels of p-Bad, Bax, Bcl-2, and Bcl-xL. *β*-actin was used as the internal controls. Three independent experiments were done. ^*∗*^p < 0.05 and ^*∗∗*^p < 0.01 versus the control group.

**Figure 5:**
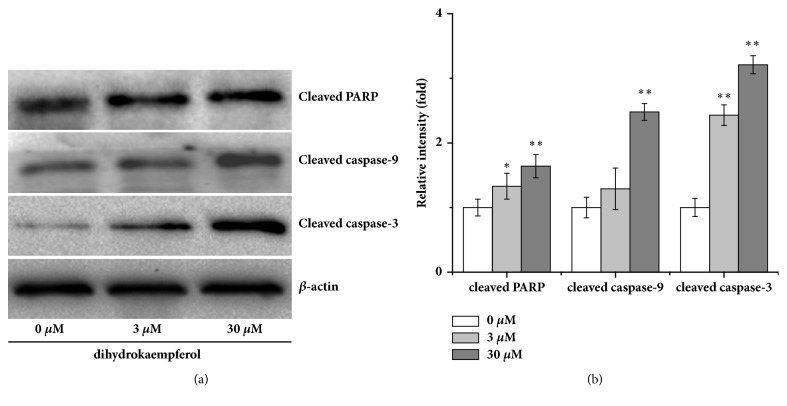
Dihydrokaempferol induced the activation of caspases and the cleavage of PARP in RA-FLSs. (a) Representative western blot and (b) the relative optical densities analysis of the levels of cleaved caspase-3, cleaved caspase-9, and cleaved PARP. *β*-actin was used as the internal controls. Three independent experiments were done. ^*∗*^p < 0.05 and ^*∗∗*^p < 0.01 versus the control group.

In further part of the study, cells were incubated with different concentrations of dihydrokaempferol to evaluate its proapoptotic activity toward synovial cells. The result (Figure 4) showed that dihydrokaempferol significantly promoted Bax and Bad expression and inhibited Bcl-2 and Bcl-xL expression. Moreover, as shown in Figure 5, the cleaved fragments of caspase-3 and caspase-9 were significantly increased by dihydrokaempferol, and the protein level of cleaved PARP was markedly increased as well. Collectively these findings indicated that dihydrokaempferol instigated apoptosis.

## 4. Discussion

*B. championii *is a traditional folk medicine which is used to treat rheumatoid arthritis. And our preliminary results [13, 14] showed that 90% EtOH extract of* B. championii* (Benth.) Benth showed a significant antiproliferative effect on synovial cells* in vitro*. To illuminate its substance foundation for pharmacologic action, in this study, a fast and efficient method for the isolation of compounds from the ethyl acetate extract of* B. championii* was developed. A total of 15 compounds were obtained, including nine flavonoids and five other compounds.

Rheumatoid arthritis is a systemic autoimmune disease associated with persistent synovitis and joint damage. Synovial cells in RA joints increase to 10-15 cell layers which grow in a tumor-like fashion. As described before, synovial cells are considered to have an important role in RA development and therefore are a perfect model in the search for new antiarthritic drugs. In the present study, to assess cell viability of the compound on RA-FLSs, the MTS reduction assay was performed. From the result of the preliminary screening, dihydrokaempferol contains antiproliferative effect on RA-FLSs. Subsequently, dihydrokaempferol was chosen in this research for further study. In light of these findings, dihydrokaempferol decreases the proliferation of RA-FLSs.

At present, apoptosis is a cascade of activation of a series of activated cell deaths after cells are stimulated by various death signals, which play an important role in maintaining many cellular functions [30, 31]. Bcl-2 family of protein is two important proteins in the process of apoptosis. Bcl-2 [32, 33] family proteins include Bcl-2, Bax, Bad, and Bcl-xL. Bcl-2 and Bcl-xL proteins inhibit apoptosis, and Bax and Bad proteins can promote apoptosis. Bcl-2 and Bax are located upstream of mitochondria and effect, finally, leading to the apoptosis of cell. In addition, Bcl-2 family exerts their pro- or antiapoptotic effect and activates the caspase family, leading to apoptosis. The caspase family is believed to play an important role in mediating various apoptotic responses [34]. Moreover, PARP is a family of proteins involved in a number of cellular processes involving mainly DNA repair and programmed cell death. It can be activated in cells experiencing stress and/or DNA damage and is inactivated by caspase cleavage. In further part of the study, dihydrokaempferol significantly promoted Bax and Bad expression and inhibited Bcl-2 and Bcl-xL expression, increasing the cleaved fragments of caspase-3, caspase-9, and the cleaved PARP. Collectively these findings indicated that dihydrokaempferol instigated apoptosis.

Compounds isolated from* B. championii* have specific characteristics; most of them are flavonoid. Flavonoid is the major component of some traditional medicinal herbs and is well known to possess a wide range of biological functions, such as antirheumatoid arthritic activity, anti-inflammation, antioxidative, and anticancer activities [35–37]. From their unique structure, flavonoid is a kind of compounds which have two benzene rings with phenol hydroxyl linking through the three carbon atoms. The parent nucleus is 2-phenyl chromones, which has the basic structure of C_6_-C_3_-C_6_. According to the structure of flavonoids, it can be further subdivided into isoflavones, flavonols, flavanones, flavanols, chalcone, and so on. Compared to kaempferol, the double bond between C-2 and C-3 is hydrogenated to single bond in dihydrokaempferol. Previous investigators have proved that kaempferol could inhibited human fibroblast-like synoviocytes proliferation and migration; furthermore, it could attenuate arthritis severity and osteoclastogenesis [38, 39]. In this study, the result showed that dihydrokaempferol demonstrated good antiproliferative effect and proapoptosis effect by promoting Bax and Bad expression and inhibiting Bcl-2 and Bcl-xL expression and initiating caspase cascade. Conclusively, these results suggested that dihydrokaempferol may be effective as a new treatment for RA and its underlying mechanism of apoptosis-promoting effect on RA-FLSs was related to caspase-dependent mitochondrial signalling pathway. Besides, the resulting effect of it with the characteristics such as the structure-activity relationships and other abilities may help to elucidate this issue or even to discover new effective agents in further studies.

*B. championii *(Benth.) Benth. is a characteristic minority medicine, which has been demonstrated to be clinically effective in treating rheumatoid arthritis (RA). In our previous studies, it was proved to be efficacious in inhibiting paw swelling and inflammations in collagen-induced arthritis (CIA) rats. Herein this paper evaluated the antiproliferative properties and the chemical components of the ethyl acetate extract from the rattan of* B. championii*, which help to understand its pharmacological material basis. It lays a sound basis for its application on RA treatment.

## 5. Conclusions

Considering the obtained results, dihydrokaempferol and thirteen other compounds were isolated from the ethyl acetate extract of* B. championii* (Benth.) Benth. Given its recorded antiproliferative effects on synovial cells, dihydrokaempferols seem to be good candidates for new antiarthritic drugs and are recommended for further biomedical studies.

## Data Availability

The data used to support the findings of this study are included within the article.
